# Evaluation of the serum tRNA-derived fragment tRF-5022B as a potential biomarker for the diagnosis of osteoarthritis

**DOI:** 10.1186/s13018-023-04273-8

**Published:** 2023-10-25

**Authors:** Yingchen Ni, Anqi Wu, Jianxin Li, Weidong Zhang, Youhua Wang

**Affiliations:** 1 Department of Orthopaedics, Affiliated Hospital of Nantong University, Medical School of Nantong University, Nantong, 226001 China; 2 Department of Thoracic Surgery, Affiliated Hospital of Nantong University, Medical School of Nantong University, Nantong, 226001 China; 3 Department of Orthopaedics, Affiliated Hospital of Nantong University, Nantong, 226001 China

**Keywords:** tRNA-derived fragments, Osteoarthritis, tRF-5022B, Biomarker, Diagnosis

## Abstract

Osteoarthritis (OA) is a degenerative disease. It is common in middle-aged and elderly people and is one of the main causes of disability. Currently, the etiology of OA is unclear, and no specific biomarkers for the diagnosis of OA have been identified. Therefore, finding a highly sensitive biomarker is essential for a proper diagnosis.

TRNA-derived fragments (tRFs) and tRNA-derived stress-induced RNAs (tiRNAs) are newly discovered classes of noncoding RNAs. tRF has been proven in several studies to have significant associations with tumor diagnosis, making it a promising biomarker in cancer research. However, the diagnostic utility of tRF in OA patients and the correlation between OA progression and trf differential expression have yet to be elaborated. The purpose of this research was to identify tRFs with differential expression in OA to assess their potential as OA biomarkers. To determine the tRF-5022B expression level in this research, real-time fluorescence quantitative PCR has been employed. Agarose gel electrophoresis, Sanger sequencing, and other investigations have been employed for evaluating tRF-5022B's molecular properties. Receiver operating characteristic curve analysis has been utilized for assessing the diagnostic effectiveness of the tRF-5022B. The findings demonstrated that tRF-5022B expression was considerably lower in OA serum. The Kellgren–Lawrence grading scale was shown to correspond with serum expression levels. The ROC curve confirmed that tRF-5022B serum expression levels might differentiate OA cases from healthy individuals and RA patients. According to the aforementioned findings, tRF-5022B may be employed as a novel biomarker for OA diagnosis due to its excellent diagnostic value.

## Introduction

Osteoarthritis (OA) is a chronic joint condition that is commonly seen in elder individuals and characterized by slowly progressive joint pain, stiffness, swelling with limited mobility, and reactive hyperplasia of subchondral bone [1, 2]. It has a series of clinical symptoms such as inflammation and bone degeneration caused by the entry of monocytes into the synovium. Chondrocytes are located on the surface the mid and deep zone of the articular cartilage tissue and can synthesize and secret matrices and fibers [3]. Therefore, the mechanism of OA has an important relationship with the function of chondrocytes. At present, the treatment of OA is divided into surgical treatment and non-surgical treatment. Non-surgical treatment includes physical exercise and drug therapy. Among them, drug treatments mainly include non-steroidal anti-inflammatory drugs and anti-inflammatory analgesics; they can only relieve symptoms but cannot block or revert the progression of treatments. The clinical diagnosis of OA is detected by plain X-ray [4]. Whether some inflammatory cytokines can act as sensitive biomarkers of OA is still under evaluation [5]. Therefore, exploring a specific biomarker to diagnose OA is essential. Early specific diagnosis can effectively prevent the progression of OA, relieve pain, and delay the need for surgeries such as joint replacement.

In recent years, noncoding RNAs, including circRNAs, microRNAs, and long noncoding RNAs (lncRNAs), have been considered as potential biomarkers of some diseases in serum [6, 7]. Transfer RNAs (tRNAs) have not been studied extensively as biomarkers. tRNAs, which range in length from 76 to 90 nucleotides, are an emerging class of noncoding RNAs (ncRNAs) [8, 9]. They have the structure of a cloverleaf, which allows them to transport amino acids to the ribosomes [10, 11]. The two major categories of tRNA-derived small RNAs (tsRNA) are tRNA-derived stress-induced RNAs (tiRNAs) and fragments (tRFs), both of which can be created by the specialized mature tRNA or tRNA precursors cleavage [12]. tRF-1 is produced by deleting the 3′ tail sequence from the tRNA precursor, tRF-5 is created by the 5′ end cleavage in the D loop, tRF-3 is obtained by the 3′ end cleavage in the T loop, and tiRNAs are obtained by the anticodon loop of mature tRNAs cleavage [13]. Nowadays, tsRNAs can be widely applied for a wide variety of biological processes, like RNA epigenetic and translational regulation and silencing [14, 15]. Also, by binding to proteins or messenger RNAs (mRNAs), tsRNAs can control gene expression [16, 17]. Numerous studies, made possible by advances in high-throughput sequencing technology, have established a crucial role for tsRNAs in the emergence of different diseases. The 3′-tRNA-derived fragment tRF-Val, for instance, binds to EEF1A1 and stimulates the growth and suppresses the death of gastric cancer cells [18]. Furthermore, tRF-3013b exerts an inhibitory effect on cell proliferation in gallbladder carcinoma by targeting TPRG1L [19]. However, current studies have not paid attention to whether tsRNA can be used as a biomarker for OA diagnosis.

In this research, we confirmed for the first time the expression of tRF-5022B in the serum of osteoarthritis patients, analyzed the correlation of serum tRF-5022B with clinicopathological features and evaluated its diagnostic efficacy. Also, tRF-5022B serum expression was demonstrated to be considerably decreased more in OA cases compared to RA cases or healthy volunteers. These all indicated it may be a useful diagnostic tool for OA diagnosis.

## Methods and materials

### Clinical specimen

Seventy OA patients' serum, twenty RA patients' serum, and seventy serum samples from healthy volunteers were obtained from the Laboratory Department of the Affiliated Hospital of Nantong University. The Ethics Committee of Nantong University's Affiliated Hospital reviewed and agreed on all study procedures before they were implemented. The consent was explained to and signed by all participants.

### Target gene prediction and bioinformatics analysis of tsRNA

Sequencing information for tRFs was downloaded from the GSE200433 repository. The miRanda, TargetScan, and RNAhybrid databases were used to predict the tRF-5022B target genes. The tRF-5022B pathway was investigated using Kyoto Encyclopedia of Genes and Genomes (KEGG) pathway analyses and Gene Ontology (GO) function analysis.

### Stability verification experiments and agarose gel electrophoresis

Twenty healthy control sera were pooled and divided equally among 10 tubes; the tubes followed by incubation at room temperature for 0, 6, 12, 18, 24 h and subjected to a series of freeze–thaw cycles (0, 1, 3, 5, 7 times) to extract RNA. The tRF-5022B Ct value was then measured. A similar dye preparation combination was applied to the gathered qRT-PCR products. In addition, a 2.5% agarose gel was made, and after 30 min of electrophoresis at 100v, the electrophoresis bands could be observed under ultraviolet light.

### Total RNA extraction and cDNA synthesis

Based on the reagent manufacturer’s protocol, the collected serum samples (300 μl) had total RNA isolation using the blood total RNA rapid extraction kit (spin column method) (BioTeke, Beijing, China). The concentration and purity of each RNA were evaluated through a spectrophotometer by determining the absorbance ratio of A260/A280, yielding values between 1.8 and 2.1. The total RNA was kept at − 80 °C or reverse transcription experiments were immediately performed. cDNA synthesis was carried out with the Revert Aid RT reverse transcription kit (Thermo Fisher Scientific).

### Quantitative reverse transcription–polymerase chain reaction (qRT–PCR) analysis

The qRT-PCR was carried as per the package directions. As a means of quality assurance, we studied U6. The formula 2^−ΔΔCt^ could be utilized for calculating the relative expression level of the sample. The experiments were repeated a minimum of three times for reliability. RiboBio (Guangzhou, China) developed and manufactured all primers utilized in this study.

### Statistical analysis

SPSS Statistics Version 20.0 (IBM SPSS Statistics, Chicago, USA) and GraphPad Prism v7.0 (GraphPad Software Inc., California, USA) were employed for analyzing the data collected. Nonparametric tests are used for data that are not normally distributed. The diagnostic utility of tRF-5022B for OA was determined by calculating the area under the receiver operating characteristic (ROC) curve and the area under the ROC curve (AUC). Results from at least three separate replicates of the tRF-5022B expression level in each group are given as means and standard deviations (SD). Statistical significance was assumed with an estimated P value of < 0.05.

## Results

### Gene bank and screening of tRF-5022B in OA

**Fig. 1:**
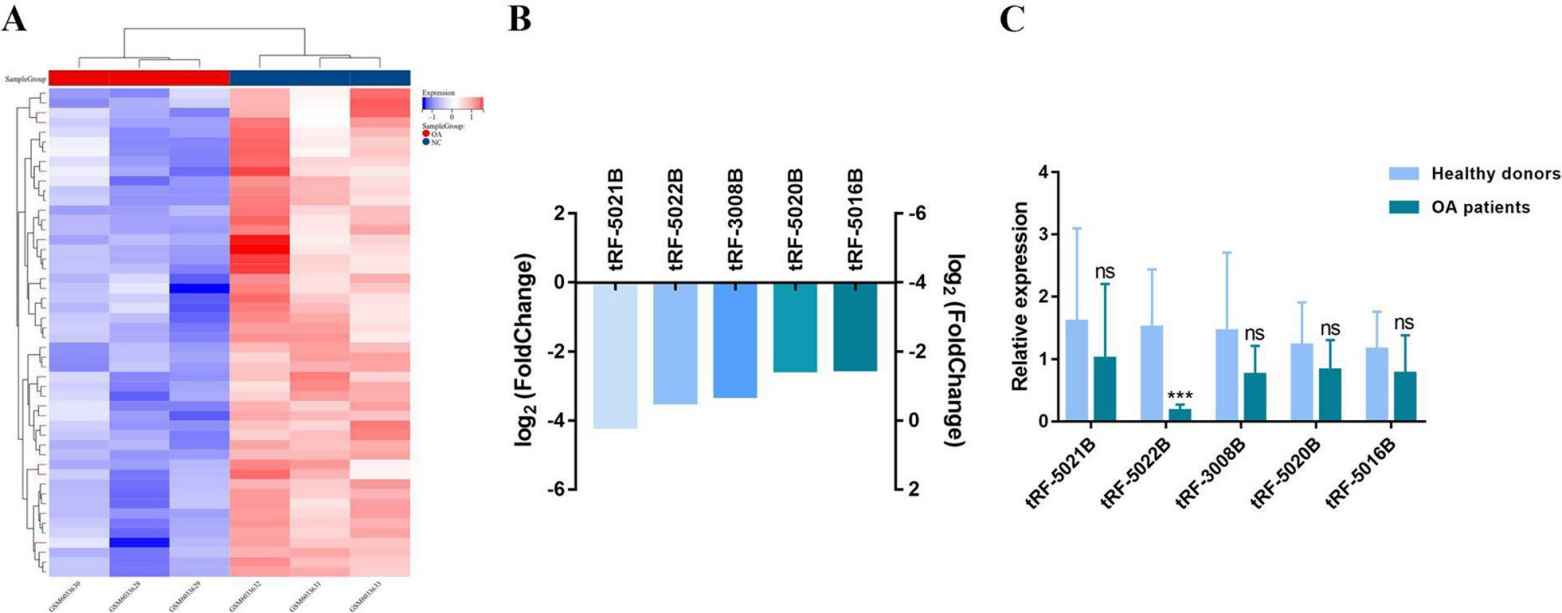
Expression profiling and screening of tsRNAs in databases. **A** cluster heatmap showing the differential expression of tsRNAs in the database. **B** Downregulated tsRNAs (*n* = 5, log2 fold change < − 1, *P* < 0.05). **C** tsRNAs expression (*n* = 5, 12 pairs of OA patient sera and healthy donor sera, ***P < 0.001)

To find a tsRNA that can serve as a good biomarker for OA, five tsRNAs whose expression levels varied significantly were selected for further consideration because their log2 fold-changes were less than -2 and the p values were less than 0.05, as seen in the heat map representing the database sequencing findings (Fig. 1A–1B). We used quantitative real-time polymerase chain reaction to identify these 5 low-expressed tsRNAs in the serum of 12 OA cases and 12 healthy controls (Fig. 1C). According to the findings, tRF-5022B expression levels varied significantly, thus we focused on this gene in the subsequent study.

### tRF-5022B is a 5′-tRF

**Fig. 2:**
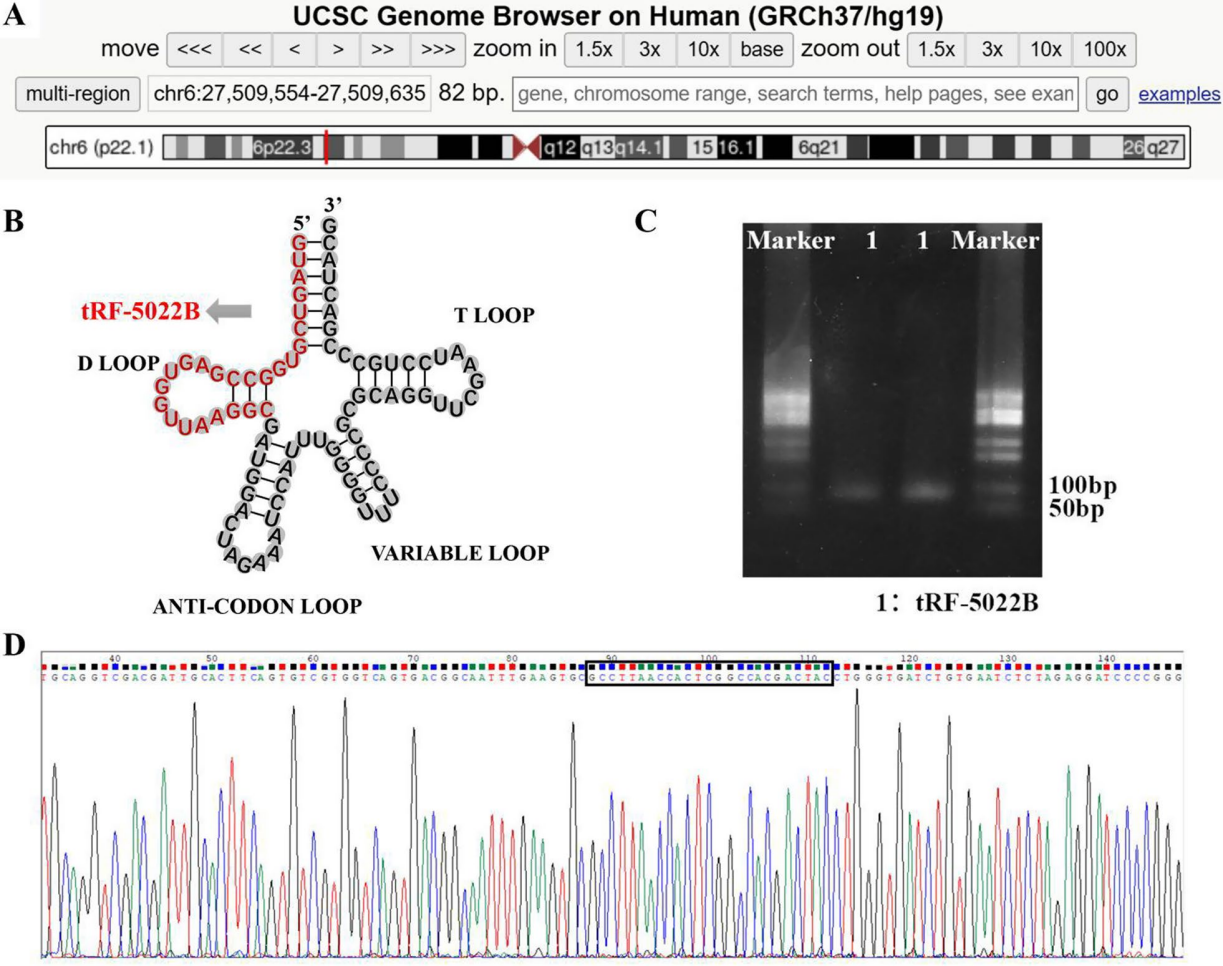
tRF-5022B is a 5′-tRF. **A** Chromosome 6P22.3 contains the tRF-5022B, with chr6:27,509,554-27,509,635 coordinates. **B** Molecular structure of tRF-5022B. **C** qRT-PCR products showed a single electrophoresis band by agarose gel electrophoresis. **D** Sanger sequencing demonstrated that the qRT-PCR product contained the full-length sequence of tRF-5022B

tRF-5022B may be found at chr6:27,509,554–27,509,635 according to the UCSC genome browser database (Fig. 2A). It has been determined that tRF-5022B is a 25nt tRF (5′- GTAGTCGTGGCCGAGTGGTTAAGGC-3′) by reviewing the OncotRF database. The sequence of tRF-5022B originates at the 5′ end of tRNA, as seen in the structural model diagram of tRF-5022B (Fig. 2B). The size of the single band of qRT-PCR product seen by agarose gel electrophoresis (AGE) was determined to be about 80 bp (Fig. 2C). The entire tRF-5022B sequence was also present, as shown by Sanger sequencing (Fig. 2D).

### Methodological evaluation of tRF-5022B

**Fig. 3:**
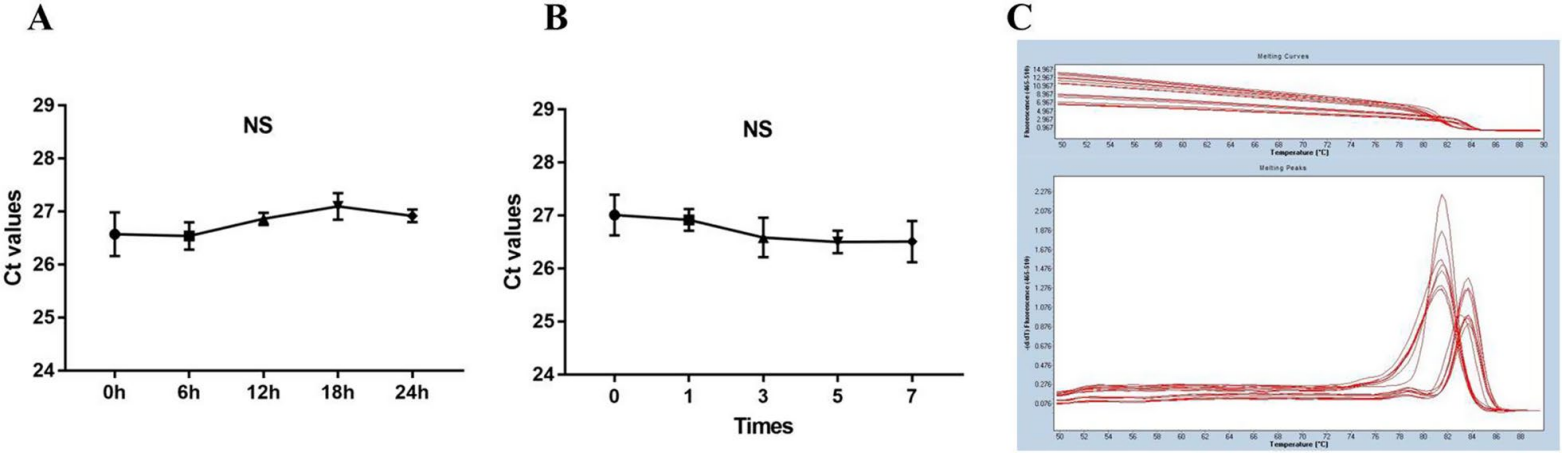
Methodological evaluation of tRF-5022B. **A** The Ct value of tRF-5022B was detected by qRT-PCR after the mixed serum was stored at room temperature for different time. **B** The Ct value of tRF-5022B was detected by qRT-PCR after the mixed serum was repeatedly frozen and thawed for different times. **C** The melting curve of tRF-5022B is unimodal and specific. NS *P* > 0.05

To determine, if tRF-5022B is suitable for clinical usage, we first subjected it to a thorough investigation. The ct value of tRF-5022B was tested using a mixture of sera from 20 healthy volunteers. The expression levels of tRF-5022B and U6 in the combined serum satisfied the criteria with respect to both intra- and inter-experimental coefficients of variation (Table 1). Aliquots of the combined serum were left for 0, 6, 12, 18, and 24 h at room temperature, frozen and thawed again at 0, 1, 3, 5, and 7 separate occasions, and finally re-tested. There was no discernible variation in tRF-5022B expression, as measured by quantitative real-time PCR (Fig. 3A, B). Indicating the reliability and consistency of the tRF-5022B detecting procedure. The melting curve of tRF-5022B was discovered to be uniquely characterized by a single peak, and its amplification curve was found to be smooth (Fig. 3C). All of these evidences suggest that clinical use of tRF-5022B detection is possible.

**Table 1:** The intra-assay CV and the inter-assay CV of tRF-5022B

	tRF-5022B	U6
Intra assay CV,%	2.27	1.94
Inter assay CV,%	2.52	2.17

### Expression and diagnostic efficacy of tRF-5022B in serum of OA

**Fig. 4:**
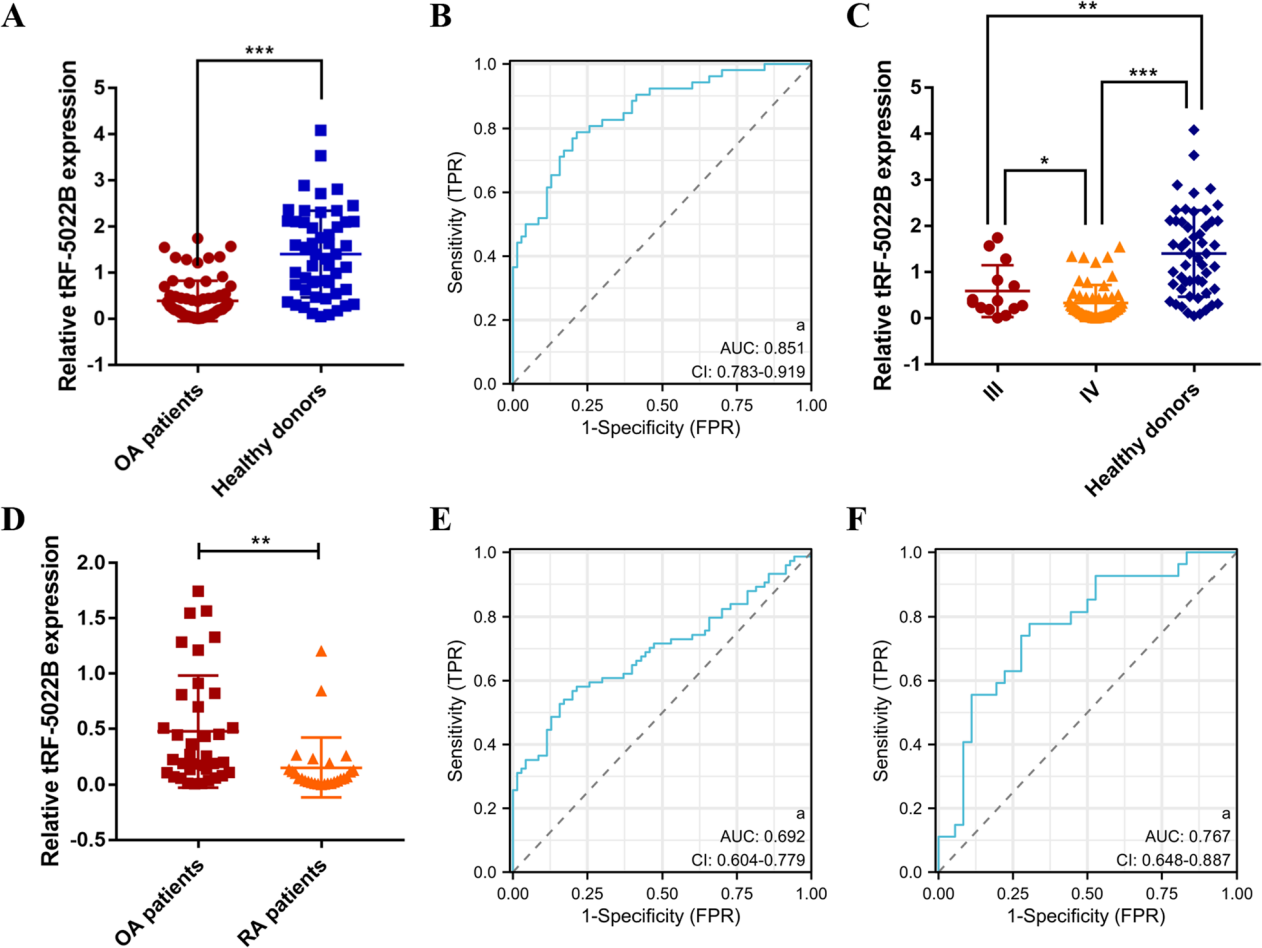
Clinical value and receiver operating characteristic (ROC) curve analysis of tRF-5022B in OA serum. **A** Expression values of tRF-5022B in OA patient and healthy donor sera. **B** ROC of the diagnostic value of tRF-5022B for OA **C** Serum tRF-5022B expression levels of different K-L stages of OA. **D** Serum tRF-5022B expression levels in OA and RA cases. **E** ROC curve analysis of tRF-5022B risk score in OA and RA patients’ sera. **F** ROC curve analysis of risk score for serum tRF-5022B in OA patients compared with all controls (RA cases and healthy donors). *P < 0.05, **P < 0.01, ***P < 0.001

We conducted a validation assessment in a separate group of 70 OA cases and 70 healthy controls to verify the serum expression level of tRF-5022B. Patients with OA had considerably lower tRF-5022B expression in their peripheral blood than did healthy controls (Fig. 4A). Based on these findings, the ROC curve for tRF-5022B's diagnostic value in OA was constructed. A value of 0.851 (95% CI, 0.783–0.919) was estimated as the AUC for tRF-5022B. The best cutoff value was found to be 0.526, and specificity and sensitivity were 78.6%, and 78.8%, respectively (Fig. 4B). As a result, serum tRF-5022B could be useful as a biomarker for OA. Serum levels of tRF-5022B were substantially higher in OA than RA cases (*P* < 0.01) (Fig. 4D). Moreover, we compared tRF-5022B levels in the blood of people with OA to those in the two control groups (those with RA and those without) to determine its diagnostic utility and 0.692 (95% CI: 0.604–0.779) was the AUC value. The best cutoff value was found to be 0.783, and specificity and sensitivity were 84.3%, and 52.7%, respectively (Fig. 4E). OA and RA patients had similar risk scores of tRF-5022B in their blood, with a value of 0.767 (95% CI 0.648–0.887) for AUC. A cutoff of 0.141 resulted in an estimated specificity and sensitivity of 69.4%, and 77.8%, respectively (Fig. 4F).

### Correlation between tRF-5022B expression and clinicopathological features

We gathered clinicopathological data from 70 OA patients to analyze the potential clinical use of serum tRF-5022B. We divided tRF-5022B expression in OA serum into two groups, high and low, according to the median value of the data. The tRF-5022B expression and clinicopathological data of 70 OA patients were analyzed using the chi-square test for significance. Table 2 shows that although there is no clear relationship between age and gender and tRF-5022B expression, there is a link between tRF-5022B expression and K-L grade (Table 2). The foregoing findings imply that low tRF-5022B expression may be important in predicting OA development. Then, we separated people into groups based on their K-L score so that we could see how tRF-5022B expression varied between the various groups and the control group. According to the qRT-PCR data, the expression of tRF-5022B was considerably reduced in patients of grades III and IV compared to healthy controls (Fig. 4C).

**Table 2:** Clinicopathological analysis of tRF-5022B

Characteristics		No. of patients	tRF-5022B (low)	tRF-5022B (high)	*p* value
Sex	Male	24	16	8	0.081
	Female	46	20	26	
Age (years)	< 65	23	10	13	0.447
	≥ 65	47	26	21	
Affected side	Left	26	9	17	0.047
	Right	44	27	17	
Disease duration (years)	< 5	37	19	18	1.000
	≥ 5	33	17	16	
Kellgren–Lawrence gradation	III	14	3	11	0.017*
	IV	56	33	23	

### Functional analysis and prediction of potential target genes of tRF-5022B in OA

**Fig. 5:**
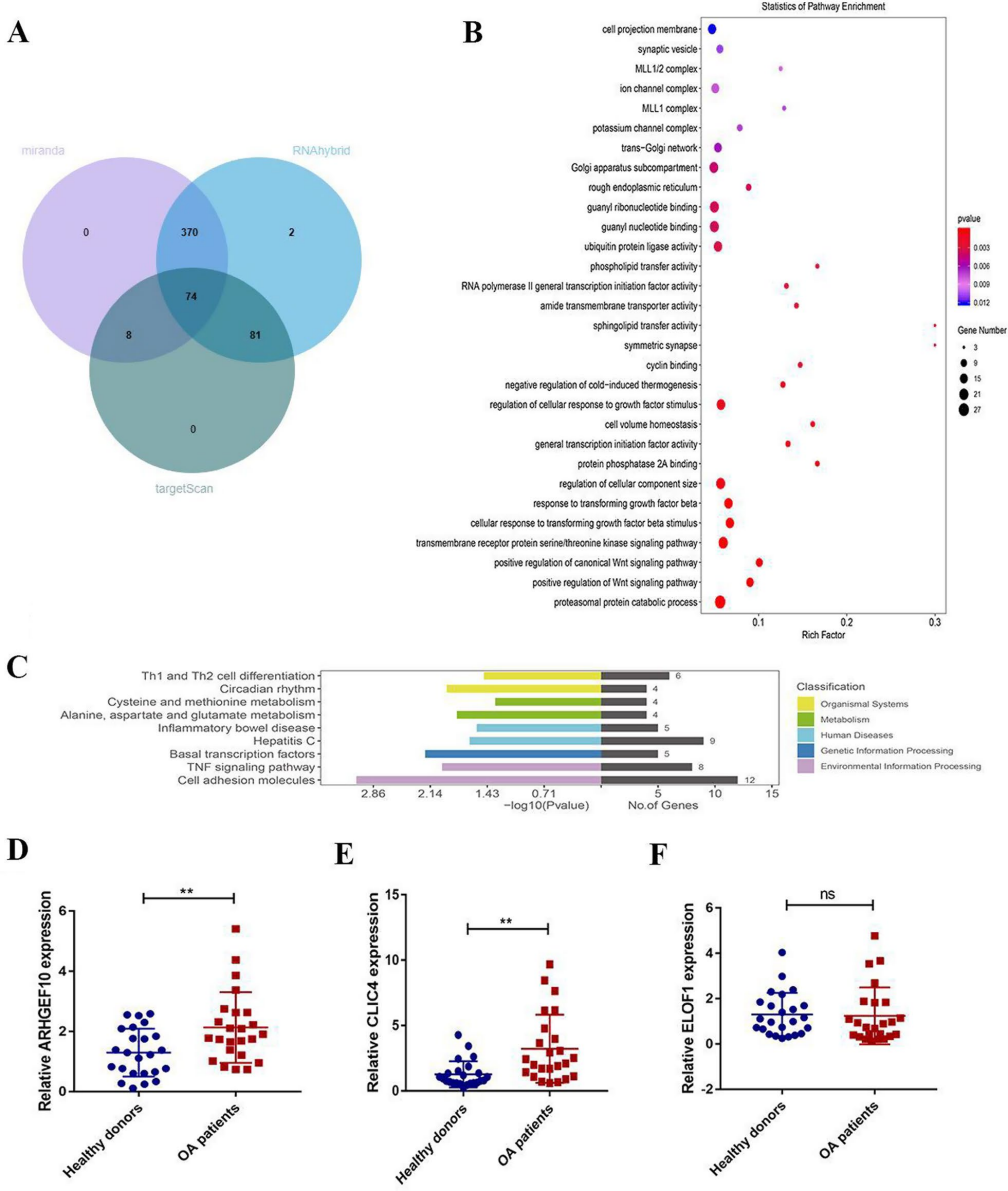
Prediction of downstream regulatory mechanism of tRF-5022B. **A** Potential target genes of tRF-5022B. **B** GO functional enrichment analysis of tRF-5022B. **C** KEGG biological pathway enrichment analysis of tRF-5022B. **D**–**E** The expression levels of ARHGEF10 and CLIC4 in OA serum were significantly higher than those in healthy donor serum. **F** There was no significant difference in the expression level of ELOF1 in OA serum and healthy donor serum. GO, Gene Ontology; KEGG, Kyoto Encyclopedia of Genes and Genomes. **P < 0.01, NS *P* > 0.05

We utilized the RNAhybrid, TargetScan, and miRanda databases to determine the downstream potential target genes of tRF-5022B in order to further investigate its mechanism of action in OA. The crossover set of 74 genes shows potential overlap with tRF-5022B's target genes (Fig. 5A). Potential target genes were also shown to be involved in the proteasomal protein catabolic process, the Wnt signaling pathway, and the transmembrane receptor protein serine/threonine kinase signaling pathway, according to GO functional enrichment analysis (Fig. 5B). According to the results of a KEGG biological pathway enrichment study, the following pathways were considerably enriched: Basal transcription factors, Cell adhesion molecules, and TNF signaling pathway (Fig. 5C). We screened three target genes (ARHGEF10, CLIC4 and ELOF1) that play roles in Wnt signaling pathway and TNF signaling pathway. We next compared the serum levels of 12 matched sets of OA patients and healthy donors to confirm the expression of three candidate target genes (ARHGEF10, CLIC4, and ELOF1). Serum levels of ARHGEF10 and CLIC4 were found to be considerably greater in OA patients compared to healthy donors (Fig. 5D, E), although ELOF1 expression did not significantly vary between the two groups (Fig. 5F). These data suggest that tRF-5022B is involved in the pathogenesis of OA by targeting downstream mRNA. However, more research is needed to determine tRF-5022B's primary regulatory mechanism in OA.

## Discussion

Osteoarthritis is a degenerative illness that causes pain and swelling in the joints over time [20–22]. However, recent research has shown that there are currently no clinically useful biomarkers for OA. And knee arthroplasty is the sole option for most patients with terminal illnesses [23]. Finding extremely sensitive biomarkers appropriate for early screening of OA is, hence, of paramount importance.

Noncoding RNAs have been shown to have a regulatory function in illness, and high-throughput sequencing has made it likely that they will serve as highly specific biomarkers in the future [24–27]. tsRNAs are a novel class of short, noncoding RNAs with promising medical applications; their structure is rather stable [28, 29]. The potential for tsRNAs to serve as biomarkers of next-generation illnesses is promising because of their abundant presence in the serum. Effects on tumor cell invasion, migration, and proliferation are only a few examples of how tsRNAs contribute to cancer's progression, which has been the subject of several research [30–32]. However, whether tsRNA can be used as a novel biomarker for OA diagnosis has not been reported.

Our research demonstrated that the low-expressed tRFs in OA were first screened in the GEO database according to log2FC and *P* value. By comparing the expressions in 12 pairs of OA cases and healthy donors’ sera, tRF-5022B had the most significant differential expression. The basic information and chromosome location of tRF-5022B were obtained according to the UCSC genome browser database information. Previous studies have demonstrated that tRFs might be employed as disease biomarkers. However, these studies lack a complete analysis of the molecular properties and methodological evaluation of tRFs. Here, we conducted a thorough methodological validation of tRF-5022B's potential as an OA biomarker. The sensitivity and reliability of the tRF-5022B have been shown. Its detection method is highly accurate and reliable in the clinical settings. Furthermore, Sanger sequencing and agarose gel electrophoresis verified the PCR amplification results. The expression level of tRF-5022B in the serum of OA patients was dramatically lowered, as shown by qRT-PCR findings after collecting a larger number of serum samples from OA patients and healthy donors. Thus, tRF-5022B has been shown to be useful in differentiating OA patients from healthy donors. We analyzed the SPSS database we built from the medical records of 70 OA patients and discovered that tRF-5022B expression level linked with Kellgren–Lawrence (K–L) grade. We also discovered that there are changes in the expression of tRF-5022B between patients with osteoarthritis and those with rheumatoid arthritis by comparing the levels of expression in their serum. As a result, tRF-5022B may also be utilized to diagnose OA and RA. The clinical utility of tRF-5022B for the diagnosis of OA has now been shown for the first time. No studies have shown a role for tRF-5022B in the pathogenesis of any other disorders. tRF-5022B has been shown to be effective in OA; however, its precise mechanism of action remains unclear. Importantly, tsRNAs have been shown to control gene expression and function by directly binding their targets. For example, tRF-17 binds THBS1 (Thrombospondin-1) and reduction in THBS1 expression rescue the effects of tRF-17 inhibition on breast cancer cell viability, invasion and migration [33]. Besides, adhesion molecule 1 (CEACAM1) as a direct target of 5'tRF-Gly was downregulated in Hepatocellular Carcinoma (HCC) and affected 5'tRF-Gly-mediated promotion of HCC cells [34]. To determine which genes tRF-5022B could be aiming after, we consulted a database. Possible signaling pathways and downstream mechanisms controlled by tRF-5022B were also predicted using GO functional enrichment analysis and KEGG biological pathway enrichment analysis. The above findings opened the path for the effect of tRF-5022B on OA development.

## Conclusions

In conclusion, we found that tRF-5022B was low expressed in serum of OA patients, which may be employed as a diagnostic biomarker in individuals with OA in clinical settings. In addition, the mechanism of action of tRF-5022B in OA needs to be further explored.

## Data Availability

The raw data supporting the conclusions of this article will be made available by the authors, without undue reservation.
